# Severe Liver Cirrhosis Markedly Reduces AhR-Mediated Induction of Cytochrome P450 in Rats by Decreasing the Transcription of Target Genes

**DOI:** 10.1371/journal.pone.0061983

**Published:** 2013-04-23

**Authors:** Maura Floreani, Sara De Martin, Daniela Gabbia, Massimo Barbierato, Alberto Nassi, Claudia Mescoli, Rocco Orlando, Sergio Bova, Paolo Angeli, Elisabetta Gola, Antonietta Sticca, Pietro Palatini

**Affiliations:** 1 Department of Pharmaceutical and Pharmacological Sciences, University of Padova, Padova, Italy; 2 Surgical Pathology and Cytopathology Unit, Department of Medicine, University of Padova, Padova, Italy; 3 Laboratory of Experimental Hepatology, Department of Medicine, University of Padova, Padova, Italy; University of Navarra School of Medicine and Center for Applied Medical Research (CIMA), Spain

## Abstract

Although the induction of cytochrome P450 (CYP) has long been investigated in patients with cirrhosis, the question whether liver dysfunction impairs the response to CYP inducers still remains unresolved. Moreover, the mechanism underlying the possible effect of cirrhosis on induction has not been investigated. Since ethical constraints do not permit methodologically rigorous studies in humans, this question was addressed by investigating the effect of the prototypical inducer benzo[a]pyrene (BP) on CYP1A1 and CYP1A2 in cirrhotic rats stratified according to the severity of liver dysfunction. We simultaneously assessed mRNA level, protein expression and enzymatic activity of the CYP1A enzymes, as well as mRNA and protein expressions of the aryl hydrocarbon receptor (AhR), which mediates the BP effect. Basal mRNA and protein expressions of CYP1A1 were virtually absent in both healthy and cirrhotic rats. On the contrary, CYP1A2 mRNA, protein and enzyme activity were constitutively present in healthy rats and decreased significantly as liver function worsened. BP treatment markedly increased the concentrations of mRNA and immunodetectable protein, and the enzymatic activities of both CYP1A enzymes to similar levels in healthy and non-ascitic cirrhotic rats. Induced mRNA levels, protein expressions and enzymatic activities of both CYPs were much lower in ascitic rats and were proportionally reduced. Both constitutive and induced protein expressions of AhR were significantly lower in ascitic than in healthy rats. These results indicate that the inducibility of CYP1A enzymes is well preserved in compensated cirrhosis, whereas it is markedly reduced when liver dysfunction becomes severe. Induction appears to be impaired at the transcriptional level, due to the reduced expression of AhR, which controls the transcription of CYP1A genes.

## Introduction

Drug interactions have became an important issue in health care, since they have often caused severe adverse effects leading to serious problems in both drug development and clinical practice [1]. Since hepatic metabolism is the major pathway of elimination of most of the drugs on the market, and cytochromes P450 (CYPs) are the most common enzymes involved, most drug interactions arise from either inhibition or induction of CYP enzymes. Although inhibition of CYPs is generally considered to be more clinically dangerous than their induction, serious or even fatal drug interactions have also been reported to occur as a result of CYP induction, e.g. transplant rejection or an exacerbation of hepatic failure following acetaminophen overdose [2], [3]. Liver function has recently been shown to play a prominent role in determining the magnitude of inhibitory drug interactions because, as liver function worsens, the degree of inhibition decreases to negligible levels [4]–[7]. On the contrary, despite over 40 years of investigation, the effect of liver disease on the magnitude of drug interactions consequent upon enzyme induction is still an unresolved question, mainly because of the highly conflicting results obtained. Thus, two reviews on this subject arrived at opposite conclusions, namely that “in severe liver disease, the ability of the liver enzymes to respond to enzyme-inducing agents is greatly curtailed” [8], and the “induction of drug metabolizing enzymes is not impaired in liver cirrhosis” [9]. Because of ethical concerns (i.e., the impossibility of administering repeated doses of a non-therapeutic inducing agent to patients with severe liver dysfunction), human studies had to rely on hepatopathic patients taking inducers for therapeutic purposes and, consequently, often lacked rigorous methodology. Although multiple factors may have contributed to the generation of conflicting results [8], a recent analysis of the literature [10] led us to conclude that these studies do not provide clear indications, mainly because they examined pathologically heterogeneous patient groups, with either unspecified degrees of liver dysfunction or including patients with mild and serious liver insufficiency.

To our knowledge, three studies addressed this question in animal models of cirrhosis. Two of these studies, which examined rats with mild liver cirrhosis, found similar degrees of induction of CYP-mediated metabolic reactions in normal and cirrhotic rats [11], [12]. A third study [13] included 5 cirrhotic rats with different degrees of liver cirrhosis, according to histological examination. The response to the inducer differed considerably in the cirrhotic group and, because of the wide scatter, this was not significantly different from that of control rats. However, the response appeared diminished in the 2 animals with more severe cirrhosis. Thus, animal investigations indicate that enzyme induction is well preserved in mild cirrhosis, whereas they do not provide any definitive conclusion as to whether it is compromised in the decompensated state of cirrhosis. Moreover, neither human nor animal studies investigated the mechanism(s) by which cirrhosis may reduce the liver response to inducing agents.

To clarify these questions, this study assessed the effect of liver dysfunction on enzyme induction in animals rigorously stratified according to the severity of liver cirrhosis. For this purpose, we used a validated animal model of liver cirrhosis produced by exposure to carbon tetrachloride (CCl_4_) [14], [15]. This method made it possible to obtain rats with compensated or decompensated cirrhosis depending on the length of exposure to CCl_4_. Control and cirrhotic rats were then treated with the prototypical inducer benzo[a]pyrene (BP), which increases primarily the expression of CYP1A1 and CYP1A2 *via* activation of the aryl hydrocarbon receptor (AhR), a transcription factor which, upon ligand binding and subsequent heterodimerization with the AhR nuclear translocator (Arnt), interacts with the AhR response element of CYP1A genes [2], [16]. The inducing effect was assessed by means of three different techniques: 1) determination of CYP1A1, CYP1A2 and AhR mRNA steady-state levels by quantitative real-time reverse transcription-polymerase chain reaction (qPCR); b) measurement of the protein expressions of CYP1A enzymes and AhR by means of Western blot analysis; c) determination of the kinetic parameters of CYP1A1 and CYP1A2 enzyme reactions by means of kinetic analysis. The simultaneous determination of the mRNA transcript, and the immunoreactive and catalytically active proteins makes it possible to discriminate between the mechanisms by which liver cirrhosis may curtail enzyme induction, i.e. by impairing gene transcription, mRNA translation or the catalytic efficiency of CYP enzymes.

## Materials and Methods

### Reagents

BP, 7-ethoxyresorufin, 7-methoxyresorufin, resorufin, NADPH, dimethylsulfoxide (DMSO), 40% acrylamide solution, sodium dodecyl sulfate (SDS), and Tween 20 were purchased from Sigma-Aldrich Italy (Milan, Italy). Phenobarbital was obtained from Bracco S.p.A. (Milan, Italy). Methanol (HPLC grade) was from Carlo Erba Reagenti (Milan, Italy). Ultrapure water was obtained by means of a Pure-Lab Option Q apparatus (Elga Lab Water, High Wycombe, UK).

Microsomes prepared from baculovirus-infected insect cells expressing rat CYP1A1 or CYP1A2 (Supersomes™) were purchased from BD Gentest (Woburn, MA, USA). The expression levels of CYP1A1 and CYP1A2 were provided by the manufacturer’s data sheets.

Mouse monoclonal antibody reactive against rat CYP1A1/2 and rabbit anti-mouse IgG used as the secondary antibody were obtained from Abcam (Cambridge, UK); mouse monoclonal antibody reactive against rat AhR was from GeneTex, Inc. (Irvine, CA, USA), and mouse monoclonal antibody reactive against rat β-actin was purchased from Santa Cruz Biotechnology, Inc. (Santa Cruz, CA, USA).

### Animals and Treatments

#### 1. Ethics Statement

The procedures involving animals and their care were in conformity with institutional guidelines that comply with national and international laws and policies (European Economic Community Council Directive 86/609,OJ L 358, 1, Dec.12, 1987; NIH Guide for the Care and Use of Laboratory Animals, NIH Publication no. 85-23, 1985). The study design was approved by the Ethics Committee of the University of Padova for the care and use of laboratory animals (Prot. no. 18758 – March 26, 2010).

#### 2. Study design

**Figure 1:**
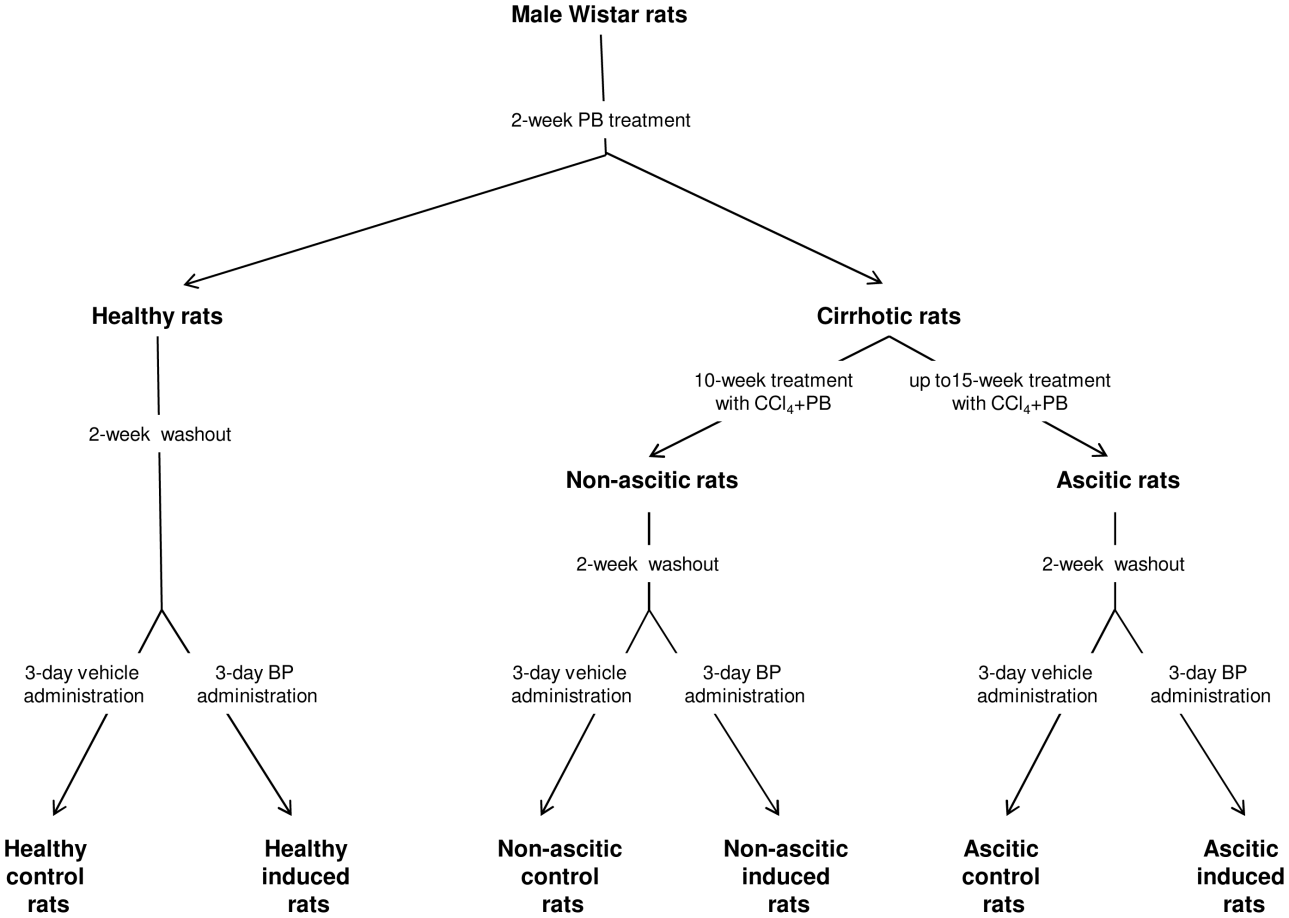
Study design. PB: Phenobarbital; BP: benzo[a]pyrene.

The investigation was performed on 60 male Wistar rats, which were divided randomly into 2 initial groups (Fig. 1): 1) Healthy rats, consisting of 16 animals; 2) Cirrhotic rats, consisting of 44 animals in which cirrhosis was induced by exposure to carbon tetrachloride (CCl_4_). Phenobarbital (0.3 g/L) was added to the drinking water of both groups for 2 weeks. After a 2-week washout period, which was shown to be sufficient to allow full withdrawal of the inducing effect of phenobarbital on hepatic CYPs [11], [13], the healthy group was divided into two subgroups: one, designated as induced healthy rats, received 20 mg/kg BP dissolved in 3 ml of corn oil once daily for 3 consecutive days by the intraperitoneal route; the other (control healthy rats) received only corn oil according to the same administration protocol. Exposure to phenobarbital in drinking water was not suspended for the other 44 rats, in which cirrhosis was induced by exposing the animals to CCl_4_ in an inhalation chamber twice a week, following a method previously described [14], [15]. Coadministration of phenobarbital greatly increases the formation rate of the reactive metabolites of CCl_4_ responsible for the development of cirrhosis, thereby drastically reducing the cirrhosis induction time [15], [17]. After 10 weeks (during which 7 rats died and 4 developed ascites, since cirrhosis progresses at different rates in CCl_4_-treated animals [13], [15]), 16 rats not showing ascites were divided into 2 groups of 8 animals. Following a 2-week washout period, these rats, which all had histologically proven cirrhosis, as ascertained from *post-mortem* histological examination (see below), were treated with BP (non-ascitic induced rats) or vehicle (non-ascitic control rats) as described above. The remaining 17 rats were further subjected to CCl_4_ treatment (which never exceeded 15 weeks) until ascites was ascertained by abdominal palpation. As ascites was ascertained, rats (including those which had developed ascites during the first 10 weeks) were alternatively treated, after a 2-week washout period, with BP (induced ascitic rats) or vehicle (control ascitic rats), as specified above. Treatments were continued until 8 ascitic rats per group were obtained.

At the end of treatment, rats were weighed and sacrificed under sevoflurane anaesthesia (2.5% per liter of oxygen) 24 hrs after the last dose of BP or corn oil. Blood was collected by cardiac puncture for biochemical tests of liver function [alanine aminotransferase (ALT), aspartate aminotransferase (AST), serum albumin and total bilirubin concentrations]. After exsanguination, livers were rapidly removed and weighed for the determination of liver index [(liver weight/body weight)×100]. A piece (3–4 g) of liver was obtained for histological examination. The liver was then rinsed in ice-cold 0.9% NaCl and a small piece (less than 100 mg of hepatic tissue) was placed in an Eppendorf tube containing 0.75 ml of TRIzol Reagent (Invitrogen, Carlsbad, CA, USA) for total RNA extraction. This sample and the remaining liver were quickly frozen and stored at −80°C until used.

### Histological Evaluation

Immediately after excision, liver specimens were fixed in 4% neutral buffered formalin for 12–24 hours. They were then grossly cut in order to obtain adequate biopsies to be embedded in paraffin. From each paraffin block at least two sections 4 µm-thick were sliced and stained with hematoxylin-eosin and picric acid-acid fuchsin (Van Gieson stain), using standard techniques. Histological examinations of liver biopsies were all performed by the same observer who was blinded from any information about the type of rat (BP-treated or untreated; healthy or cirrhotic). Images were obtained by means of a Leica SCN400 slide scanner. In order to evaluate the degree of the CCl_4_-induced liver damage, the Ishak scoring system was adopted, which scores livers from 0 to 6 according to the severity of cirrhotic alterations [18].

### Preparation of Liver Microsomal Fraction

Frozen hepatic tissue (about 7–8 g) was allowed to thaw in ice-cold homogenizing buffer consisting of 50 mM Tris-HCl, 150 mM KCl and 2 mM EDTA (pH 7.4). After complete thawing, each liver sample was quickly dried, weighed, dissected into small pieces, placed in a Potter-Elvehjem-type glass mortar in the presence of 2 volumes (w/v) of ice-cold homogenizing buffer and homogenized in ice for two 5-sec intervals with an IKA T25 Ultra-Turrax disperser (IKA Werke GmbH & Co., Staufen im Breisgau, Germany) provided with a S25N-18G dispersing tool. This suspension, further homogenized with a motor-driven tissue homogenizer and diluted with 3 volumes of the homogenizing buffer, was then processed according to the method described by Pearce *et al*. [19] to obtain the microsomal fraction.

Protein concentration in the microsomal fraction was determined by means of a commercial available kit (Novagen BCA Protein Assay kit) using a standard calibration curve obtained with known amounts of bovine serum albumin.

Microsomal total CYP content was evaluated from the CO difference spectrum, as described by Omura and Sato [20].

### Total Protein Extraction from Hepatic Tissue

For Western blot analysis of AhR expression in hepatic tissue, total protein extraction was performed from liver samples by homogenization in a modified Radio-Immunoprecipitation Assay (RIPA) buffer consisting of 25 mM Tris-HCl (pH 7.4), 150 mM NaCl, 1 mM EDTA, 1% Triton-X-100, 1% sodium deoxycholate, 0.1% SDS, and a Complete Protease Inhibitor Cocktail (Roche, Milan, Italy). For this purpose, frozen hepatic tissue (about 0.2 g) was allowed to thaw in ice-cold RIPA buffer and then homogenized in 10 volumes (w/v) of the same buffer in ice for two 5-sec intervals with an IKA T25 Ultra-Turrax disperser. The homogenate was centrifuged at 4°C for 15 min at 250×*g*, and the supernatant was collected and further centrifuged at 4°C for 5 min at 2200×*g*. The final supernatant, referred to as “total liver protein extract”, was assessed for its protein content by means of the Novagen BCA Protein Assay kit.

### Determination of mRNA Levels by qPCR

Total RNA was isolated from liver samples by means of the TRIzol Reagent according to the manufacturer’s instructions. RNA integrity and quantity were determined by an RNA 6000 Nano assay in an Agilent BioAnalyser (Agilent Technologies Inc., Palo Alto, CA, USA). Reverse transcription (RT) was performed by means of Superscript III reverse transcriptase with 250 ng of total RNA and random oligonucleotides (Invitrogen).

Real-time RT-PCR was performed as previously described [21], using the same primer sequences. The expression levels of the target genes (*CYP1A1*, *CYP1A2*, *AhR*) and the housekeeping gene (*β-actin*) were quantified by means of the relative standard curve method. The expression of target genes was normalized to that of the *β-actin* gene and modifications of mRNA levels were expressed as fold variation compared with that of control healthy rats.

### Western Blot Analysis

The analysis of CYP1A1, CYP1A2 and AhR protein expression was performed as previously described [21]. Briefly, 10 µg per lane of hepatic microsomal proteins from uninduced rats, 0.5 µg of microsomal proteins from BP-treated rats, as well as rat recombinant CYP1A1 (0.1 pmol) and CYP1A2 (0.25 pmol) enzymes as reference standards, were subjected to sodium dodecylsulfate polyacrylamide gel electrophoresis (SDS-PAGE) on 10% polyacrylamide gels according to Laemmli [22].

Signal intensity of immunoreactive bands was analyzed by the Quantity One software (Bio-Rad Laboratories S.r.l.) and was expressed as intensity * mm^2^ (INT*mm^2^). Calibration curves were generated by plotting INT*mm^2^ of the reference standards (recombinant CYP1A1 and CYP1A2 enzymes) against the amount (pmol) of recombinant enzyme loaded. The amount of each CYP enzyme per lane was calculated relative to the calibration curve. The specific amount of CYP1A1 and/or CYP1A2 contained in each microsomal preparation was calculated by dividing the amount of enzyme per lane by the amount of microsomal proteins loaded per lane and was reported as pmol CYP1A1 or CYP1A2/mg protein.

For the evaluation of AhR expression in liver samples from control and BP-treated rats of all groups, total liver protein extract (60 µg of proteins per lane) was used. Proteins were resolved by SDS-PAGE on 8% polyacrylamide gels, as previously described [21]. Since recombinant AhR was not available, the signal intensity (INT*mm^2^) of the AhR immunoreactive band was normalized to that of the β-actin band, and results were expressed as fold variation with respect to control healthy rats.

### Determination of O-dealkylation of Ethoxyresorufin (EROD Activity) and Methoxyresorufin (MROD Activity) by Liver Microsomes

The incubation mixture (total volume: 0.4 ml) contained 0.1 M KH_2_PO_4_ (pH 7.4), 0.5 mM NADPH, increasing concentrations of ethoxyresorufin or methoxyresorufin (from 0.1 to 5 µM, *n* = 7), and microsomal proteins. For each microsomal preparation preliminary experiments were performed, at the lowest and highest substrate concentrations used for the kinetic assays, in order to assess linearity with respect to incubation time and microsomal protein concentration. For the assay of EROD activity, incubation time was always 5 min, whereas protein concentration had to be varied according to the activity of the microsomal preparation; this ranged from 1 µg/ml (microsomes from BP-induced healthy animals) to 250 µg/ml (microsomes from non-induced ascitic rats). For MROD activity, incubation time was 10 min, and protein concentration ranged from 12.5 µg/ml (induced healthy animals) to 250 µg/ml (non-induced ascitic rats). After a 3-min thermal equilibration at 37°C, the enzyme reactions were started by the addition of NADPH and, after shaking in a water bath at 37°C in aerobic conditions, they were stopped by adding 0.4 ml ice-cold methanol and cooling the sample in ice. The mixture was then centrifuged for 10 min at 20000×*g* (4°C) to remove denatured proteins. An aliquot (60 µl) of the supernatant was analyzed for resorufin quantification by means of a modification [21] of the HPLC method with fluorescence detection, as originally described by Hanioka *et al.* [23]. Quantitative determination of resorufin was carried out by standard calibration curves obtained with authentic resorufin at concentrations ranging from 0.0025 to 0.08 nmol/0.4 ml (*n* = 10), processed in exactly the same way as the samples obtained from kinetic experiments. The calibration curves were linear in this concentration range (*r* ^2^≥0.99), the lowest value of the range representing the limit of quantification of the assay. Both inter- and intra-assay CVs for resorufin determination (n = 5) were lower than 5% at 0.0025 nmol/0.4 ml and lower than 3% at 0.08 nmol/0.4 ml. EROD and MROD activities were expressed as pmol of resorufin produced per mg of protein per min.

#### Kinetic and statistical analyses

Initial velocity (v) data for resorufin formation catalyzed by rat liver microsomes in the presence of ethoxyresorufin (EROD activity) or methoxyresorufin (MROD activity) were evaluated by best-fitting procedures, by means of the GraphPad Prism software, version 5 (GraphPad Software Inc., San Diego, CA, USA). The F test was used to discriminate between different kinetic models (one- or two-enzyme Michaelis-Menten model, or one-enzyme Michaelis-Menten kinetics with substrate inhibition). Kinetic parameters were estimated by non-linear regression analysis of untransformed initial velocity data (GraphPad Prism software) using the appropriate equations [24]. The following kinetic parameters were determined: V_max_, maximum velocity of the reaction; K_m_, substrate concentration yielding 50% of V_max_.

Statistical analyses were also performed by means of the GraphPad Prism software. Unless otherwise indicated, the data are presented as mean values ± S.E. Comparison of the experimental data obtained from healthy, non-ascitic and ascitic cirrhotic groups was made by one-way analysis of variance (ANOVA). In case of significant differences (α = 0.05), the analysis of variance was followed by Newman-Keuls post-hoc test. Comparison of the results obtained from control and induced animals within each group was performed by means of Student’s t-test. P<0.05 was considered statistically significant.

## Results

### Serum Chemistry, Histology and Microsomal Total CYP Content of Rat Livers

**Figure 2:**
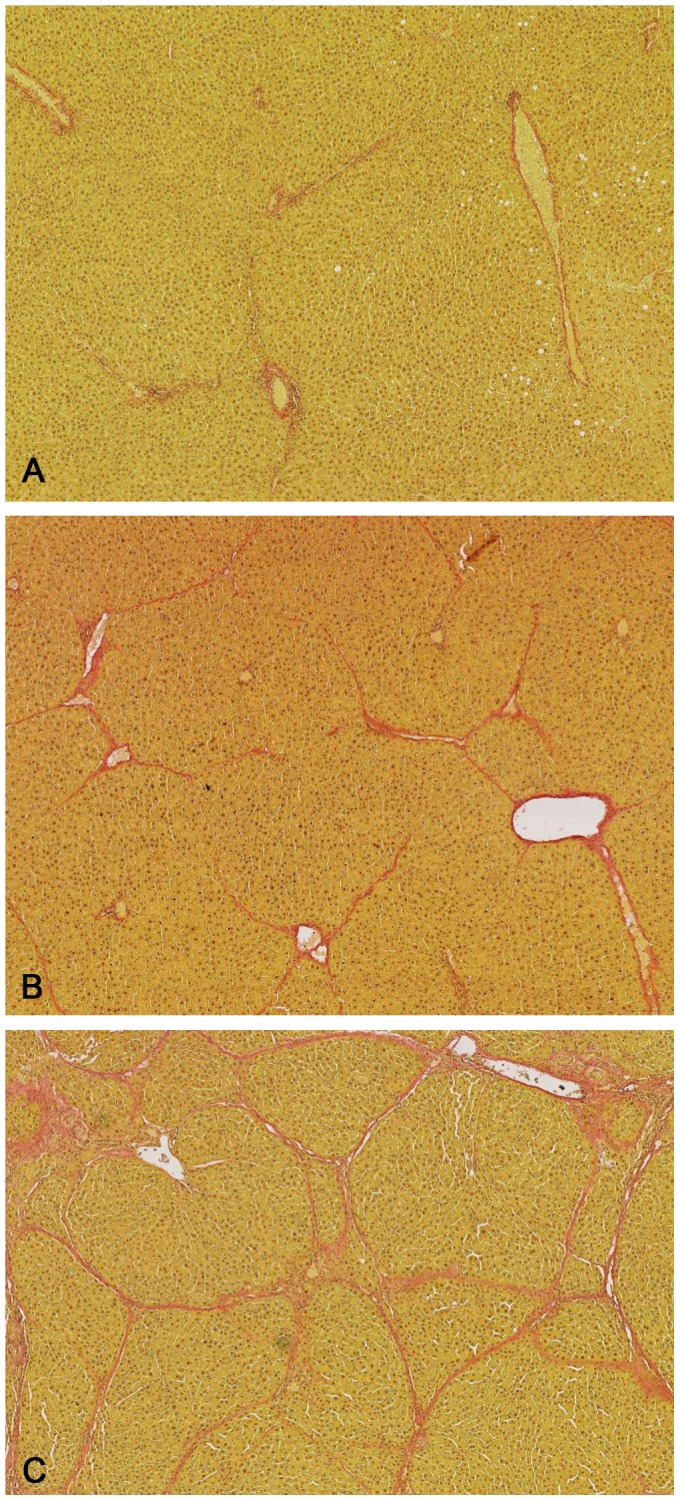
Histology of rat livers. Representative photomicrographs (Van Gieson stain, 50×magnification) of liver sections taken from an healthy (Ishak score = 0) (A), a non-ascitic (Ishak score = 4) (B), and an ascitic cirrhotic rat (Ishak score = 6) (C).

The results of biochemical tests of liver function and histological examination of healthy and cirrhotic rats are shown in Table 1 and Fig. 2. Apart from serum albumin, there were only minor, statistically non-significant differences between healthy and non-ascitic cirrhotic rats. Liver index was increased only in BP-treated rats. On the contrary, in rats with ascites all indexes of liver function were significantly altered, indicating advanced hepatocellular insufficiency. Histological examination revealed normal liver architecture in healthy animals (Fig. 2A). Livers from non-ascitic rats (Fig. 2B), despite having still recognizable liver architecture, showed fibrous septa, which were never present in healthy animals. Livers from ascitic animals showed bridging fibrosis surrounding cirrhotic nodules, with the original liver structure completely destroyed (Fig. 2C).

**Table 1:** Biochemical tests of liver function, histological examination and microsomal total CYP content.

Liver tests	HealthyControl	HealthyBP-treated	Non-asciticControl	Non-asciticBP-treated	AsciticControl	AsciticBP-treated
AST (U/L)	126±21	131±12	151±23	146±49	306±75*** ^,^ §§§	423±127 *** ^,^ §§§
ALT (U/L)	40±6	35±5	62±6	61±18	99±40*** ^,^ §§	130±51*** ^,^ §§§
Total Bilirubin (µM)	1.45±0.40	1.10±0.60	1.07±0.60	0.70±0.30	2.59±1.26* ^,^ §§	4.13±1.30*** ^,^ §§§
Albumin (g/L)	40.2±2.8	38.0±2.3	35.0±1.2***	33.8±1.1**	30.2±2.0*** ^,^ §§§	28.0±5.7*** ^,^ §§
Liver index (%)	2.83±0.38	3.03±0.34	3.21±0.41	3.68±0.59*	3.75±0.61** ^,^ §	4.44±0.78*** ^,^ §
Ishak score (0–6)(median and range)	0 (0–0)	0 (0–0)	4 (3–5)	4 (3–5)	6 (6–6)	6 (6–6)
Total CYP content(nmol/mg protein)	0.61±0.10	0.94±0.15^c^	0.64±0.07	0.88±0.14^b^	0.48±0.05** ^,^ §§	0.53±0.11*** ^,^ §§§

Results are means ± S.D. of data obtained from 8 rats per group.

* P<0.05,

** P<0.01,

*** P<0.001 *vs.* healthy rats;

§ P<0.05,

§§ P<0.01,

§§§ P<0.001 *vs.* non-ascitic cirrhotic rats;

b P<0.01,

c P<0.001 *vs*. control rats of the same group. Apart from total CYP content, results obtained from control and BP-treated animals within each group were not significantly different (P>0.05).

Table 1 also shows that the total CYP content of the microsomal fraction obtained from non-ascitic cirrhotic rats was similar to that of healthy animals, whereas that of ascitic rats was significantly decreased. Although BP induces only the enzymes of the CYP1 family, total CYP content was significantly increased in BP-treated healthy and non-ascitic cirrhotic rats, whereas no significant increase was observed in rats with ascitic cirrhosis.

### mRNA and Protein Expression of CYP1A1 and CYP1A2

**Figure 3:**
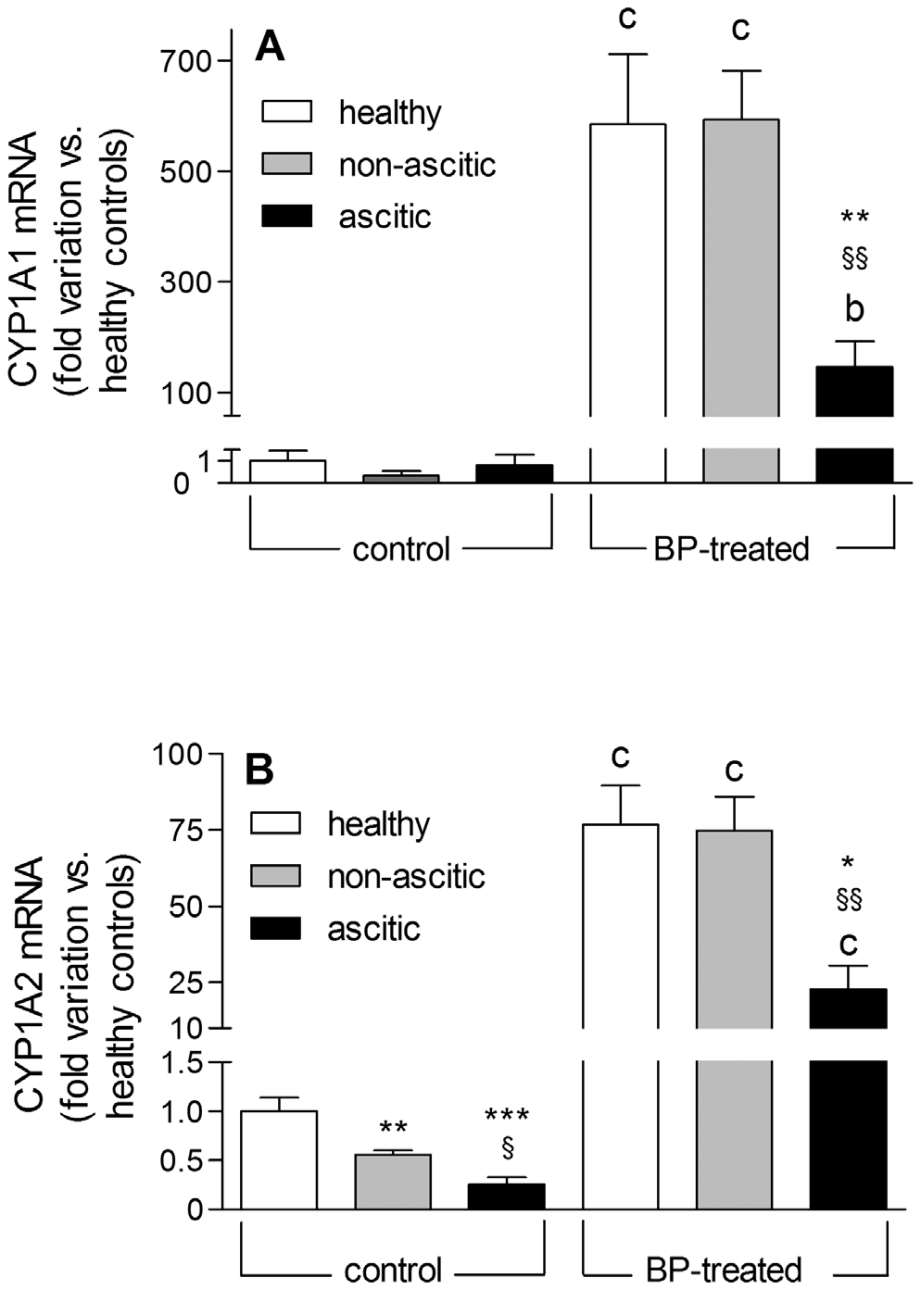
*CYP1A1* and *CYP1A2* gene transcription levels in control and BP-treated rats. The experimental data for CYP1A1 (A) and CYP1A2 (B) mRNA levels are reported as fold variation compared with healthy control rats. Results are means ± S.E. of data obtained from 8 rats per group. *P<0.05, **P<0.01, ***P<0.001 *vs.* healthy rats. ^§^P<0.05, ^§§^P<0.01 *vs.* non-ascitic cirrhotic rats. ^b^P<0.01, ^c^P<0.001 *vs.* control rats of the same group.

Fig. 3 shows the values of CYP1A1 and CYP1A2 mRNA expression relative to that of control healthy rats. CYP1A1 mRNA was present at extremely low levels in uninduced rats and increased dramatically in BP-induced healthy and non-ascitic cirrhotic animals. On the contrary, a significantly lower induction was observed in ascitic rats (Fig. 3A). At variance with CYP1A1, a significant level of CYP1A2 mRNA was present in uninduced healthy rats (about 500 times higher than that of CYP1A1), which decreased markedly with increasing severity of liver cirrhosis (Fig. 3B). The induction pattern was quite similar to that observed for CYP1A1, since CYP1A2 mRNA was increased to a significantly lower level in ascitic rats, compared with healthy and non-ascitic animals.

**Figure 4:**
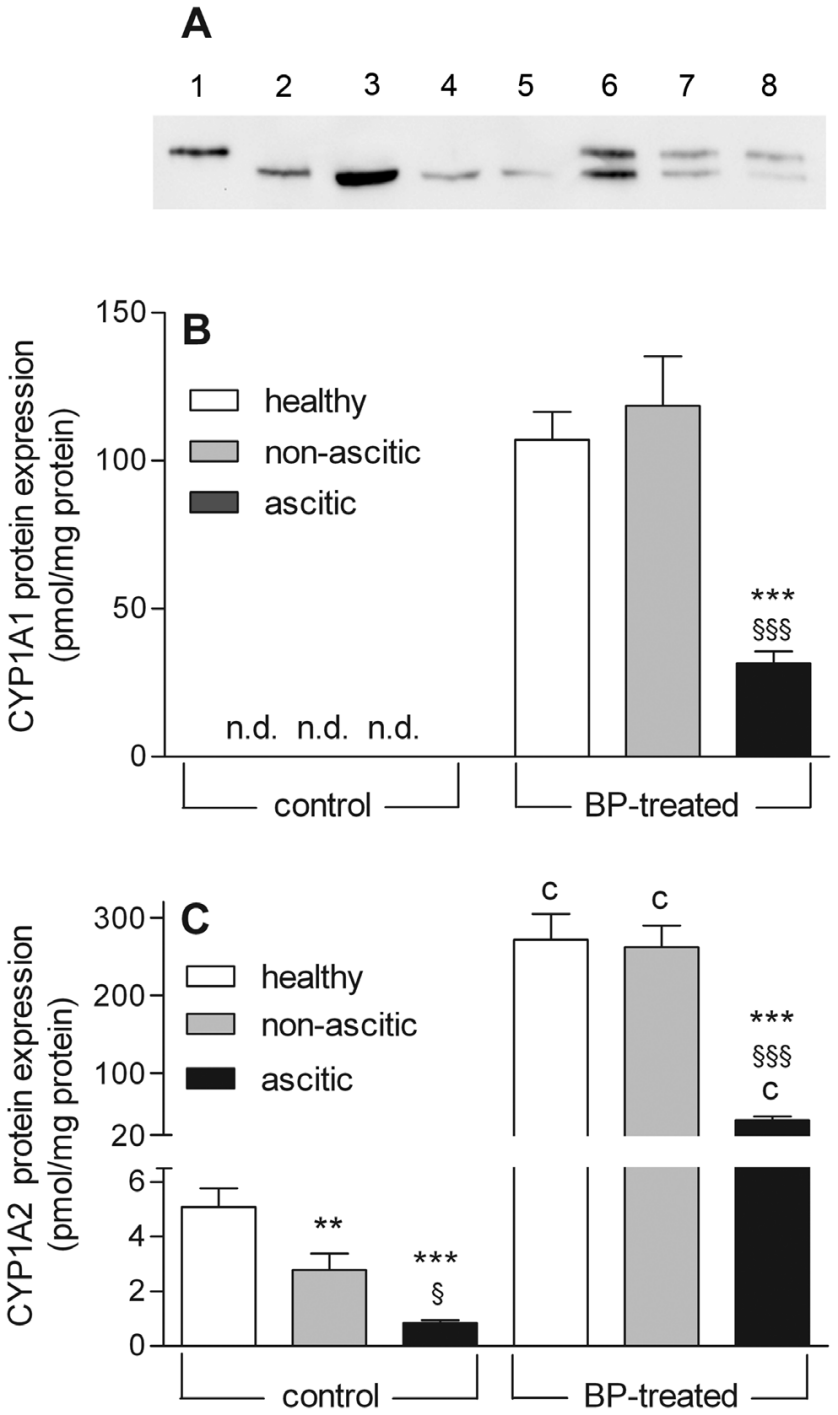
Western blot analysis of CYP1A1 and CYP1A2 proteins. (A) Representative blot showing the separation of microsomal proteins obtained from control and BP-treated rats. The amounts of microsomal proteins loaded per lane were 10 and 0.5 µg for control and BP-treated rats, respectively. As reference standards, 0.1 pmol of rat recombinant CYP1A1 protein and 0.25 pmol of rat recombinant CYP1A2 protein were loaded in lanes 1 and 2, respectively. Lanes 3 to 5: healthy, non-ascitic and ascitic control rats; lanes 6 to 8: healthy, non-ascitic and ascitic BP-treated rats. *Lower Panels*: Levels of CYP1A1 (B) and CYP1A2 (C) proteins present in liver microsomal preparations obtained from control and BP-treated rats. The results are shown as means ± S.E. of data obtained from 8 rats per group. **P<0.01, ***P<0.001 *vs.* healthy rats. ^§^P<0.05, ^§§§^P<0.001 *vs.* non-ascitic cirrhotic rats. ^c^P<0.001 *vs.* control rats of the same group.

Western blot analysis (a representative Western blot is shown in Fig. 4A) provided results that were fully consistent with those of qPCR determination: in liver microsomes of control rats (lanes 3–5) CYP1A1 protein was undetectable, whereas CYP1A2 was constitutively expressed at a considerable level and decreased significantly as liver cirrhosis progressed. In microsomal preparations obtained from induced rats (lanes 6–8) both CYP1A1 and CYP1A2 proteins were clearly evident. The difference in CYP1A2 content between microsomal preparations from BP-treated and untreated animals was actually much greater that that appearing from Fig. 4A, since 20-fold larger amounts of microsomal proteins from uninduced animals had to be loaded per lane. Normalization of signal intensity to the amount of protein loaded per lane showed that CYP1A1 and CYP1A2 were induced to similar levels (Figs. 4B and C) in healthy and non-ascitic cirrhotic rats, whereas their induction was significantly lower in rats with ascites. The relative increase in CYP1A2 protein content was 50 times in the first two groups of rats, and 7 times in the last group.

### AhR mRNA and Protein Expression

**Figure 5:**
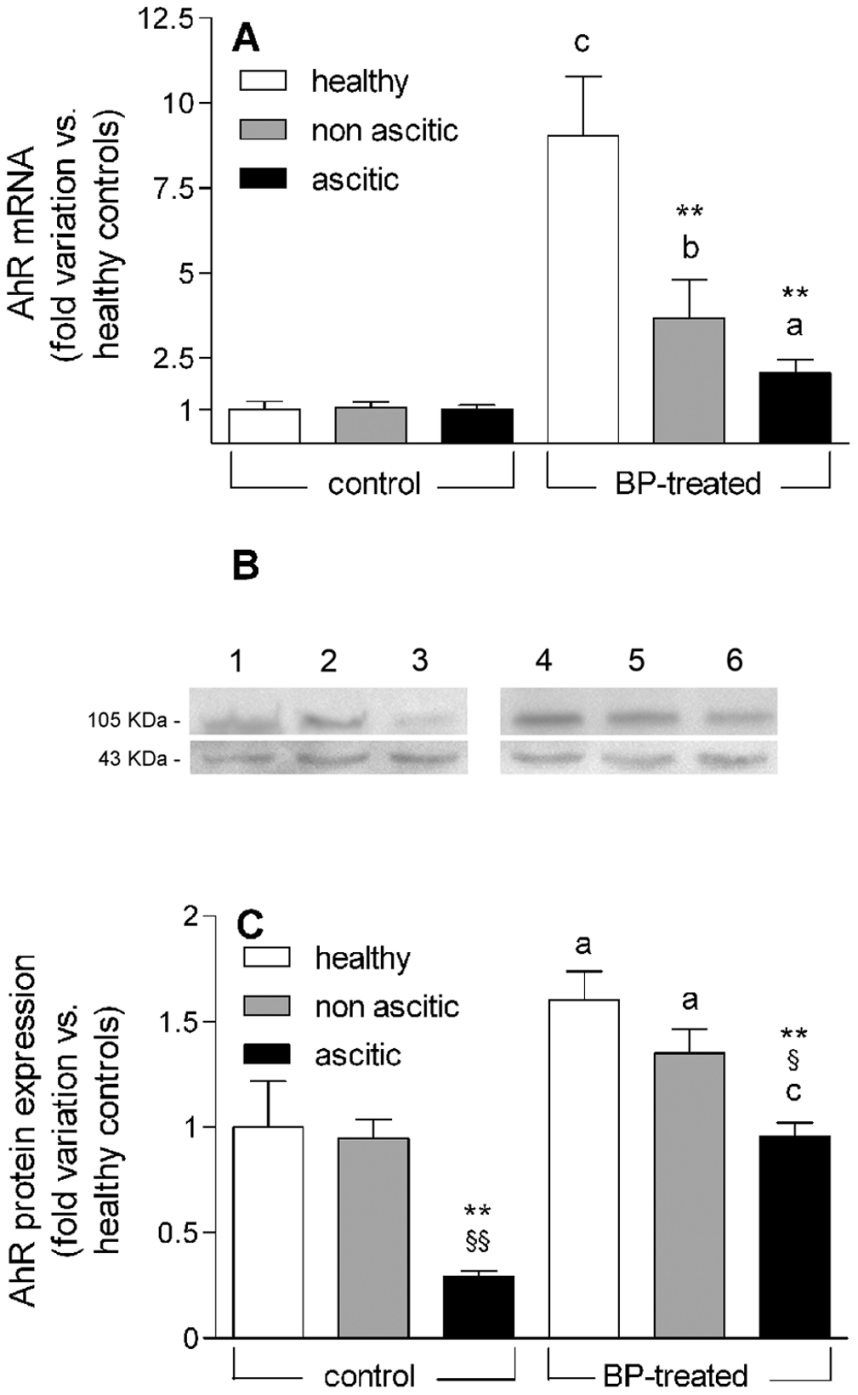
*AhR* gene transcription levels and protein expression in control and BP-treated rats. (A) Experimental data showing AhR mRNA levels are reported as fold variation compared with healthy control rats. (B) Representative Western blots showing the separation of total liver protein extracts obtained from control and BP-treated rats. The amounts of proteins loaded per lane were 60 µg. The ticks indicating 105 and 43 KDa correspond to the AhR and β-actin bands, respectively. Lanes 1 to 3: healthy, non-ascitic and ascitic control rats; lanes 4 to 6: healthy, non-ascitic and ascitic BP-treated rats. (C) Amount of AhR protein in livers from control and BP-treated rats normalized to the signal intensity of the corresponding β-actin band and shown as fold variation compared with healthy control rats. All results are means ± S.E. of data obtained from 8 rats per group. **P<0.01 *vs.* healthy rats. ^§^P<0.05, ^§§^P<0.01 *vs.* non-ascitic cirrhotic rats. ^a^P<0.05, ^b^P<0.01, ^c^P<0.001 *vs.* control rats of the same group.

Since induction of CYP1A enzymes by BP is mediated by the Ah receptor, both the mRNA level and protein content of AhR were measured. Fig. 5A shows that the constitutive levels of AhR mRNA were similar in healthy and cirrhotic rats, whereas induced levels were considerably lower in cirrhotic animals. At variance with qPCR determinations, Western blot analysis (representative Western blots are shown in Fig. 5B) revealed that the basal expression of the AhR protein was markedly reduced in ascitic rats. Fig. 5C confirms that AhR protein expression in control rats was partially dissociated from mRNA levels, since basal AhR protein content was significantly lower in ascitic rats. However, the induction pattern was qualitatively similar to that observed for mRNA (Fig. 5A).

### Kinetic Analysis of EROD and MROD Activities of Microsomal Preparations

**Figure 6:**
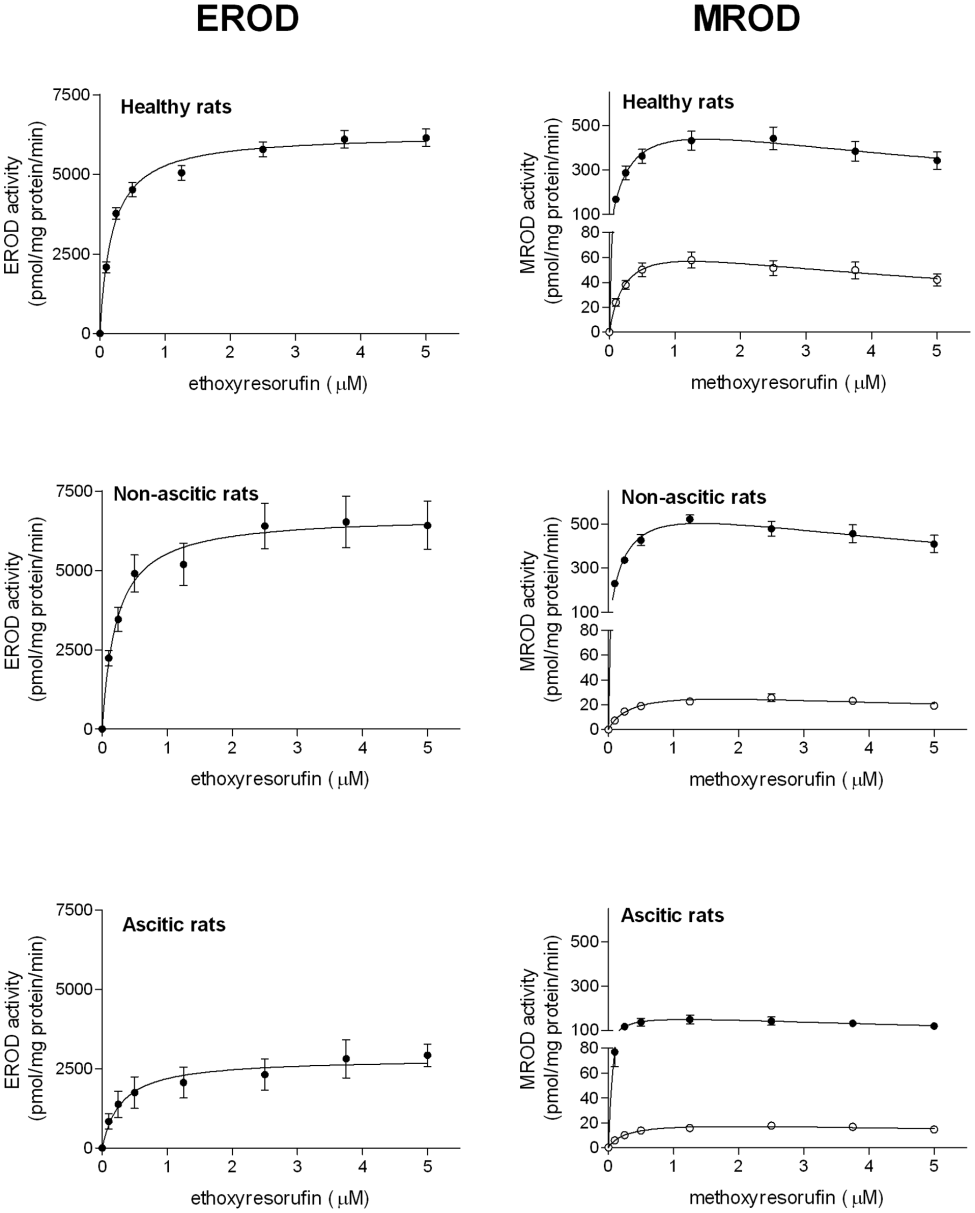
Kinetics of EROD and MROD activities in liver microsomes. Microsomal activities of control (○)BP-treated (•) rats. Results are means ± S.E. of data obtained from 8 rats per group. For each rat, activity determinations were performed in triplicate. S.E. is not shown where the size of data points is larger than the S.E. bar.

The kinetics of EROD activity, a marker reaction for CYP1A1 in BP-induced rats [21], [25], was studied, under rigorously controlled initial-rate conditions, by determining the rate of resorufin formation at increasing concentrations of ethoxyresorufin. Untransformed initial velocity data were best fitted by the equation describing one-enzyme Michaelis-Menten kinetics. Although CYP1A1 protein was absent in microsomes obtained from uninduced rats (Fig. 4A and B), a significant EROD activity was observed, but was not reported in Fig. 6 and Table 2, because it is catalyzed by other CYP forms (see Discussion). Following BP treatment, EROD activity was considerably higher in healthy and non-ascitic rats compared with ascitic animals (Fig. 6). Consistent with these observations, Table 2 shows that in induced animals the V_max_ value of the reaction was significantly higher in healthy and non-ascitic cirrhotic rats than in rats with ascitic cirrhosis. K_m_ values were very similar in all groups of BP-treated rats indicating that the same enzyme was responsible for ethoxyresorufin dealkylation in healthy and cirrhotic animals.

**Table 2:** Kinetic parameters for EROD and MROD activities of microsomal preparations obtained from control and BP-treated rats healthy and cirrhotic rats.

Kinetic parameters	HealthyControl	HealthyBP-treated	Non-asciticControl	Non-asciticBP-treated	AsciticControl	AsciticBP-treated
**EROD activity**						
V_max_ (pmol/mgprotein/min)	−	6282±339	–	6657±839	–	2674±249*** ^, §§§^
K_m_ (µM)	–	0.20±0.02	–	0.21±0.02	–	0.20±0.04
**MROD activity**						
V_max_ (pmol/mgprotein/min)	82.63±7.32	635.22±47.08^c^	40.79±1.79***	658.15±37.53^c^	26.48±1.42*** ^,^ §	184.30±13.17*** ^,§§§,^ c
K_m_ (µM)	0.26±0.01	0.29±0.03	0.29±0.05	0.27±0.03	0.28±0.05	0.28±0.05

Results are means ± S.E. of data obtained from 8 rats per group. In parenthesis the number of rats belonging to each group. Comparison of the data obtained from healthy, non-ascitic and ascitic cirrhotic groups was made by one-way analysis of variance (ANOVA), followed by Newman-Keuls post-hoc test.

*** P<0.001 *vs.* healthy rats;

§ P<0.05, and ^§§§^P<0.001 *vs.* non-ascitic rats. Comparison of the results obtained from control and BP-treated animals within each group was performed by means of Student’s t-test.

c P<0.001.

With microsomes from all groups of rats, initial velocity data of MROD activity (marker reaction for CYP1A2 [21], [25]) were best fitted by the one-enzyme Michaelis-Menten equation with substrate inhibition (Fig. 6). This figure clearly shows that basal activity decreased as liver function impairment increased and that induced activity was markedly lower in ascitic rats. Table 2 consistently shows that in uninduced rats V_max_ decreased significantly with the progression of liver cirrhosis. Like EROD activity, V_max_ of MROD activity increased to similar levels in BP-treated healthy and non-ascitic rats, and to a significantly lower level in rats with ascites. K_m_ values were very similar in all groups of rats, indicating that the same enzyme catalyzed MROD activity in untreated and BP-treated rats.

## Discussion

This study has employed for the first time an animal population rigorously stratified according to the severity of liver cirrhosis to evaluate the effect of liver dysfunction on the inducibility of hepatic CYP enzymes. This is also the first study which has set out to elucidate the mechanism underlying the effect of cirrhosis on enzyme induction by simultaneously assessing the mRNA levels, protein expressions and enzymatic activities of the induced enzymes, as well as the mRNA and protein expression of the nuclear receptor which mediates their induction. For this purpose, the AhR-mediated induction of CYP1A enzymes has been investigated, since the molecular machinery of this type of induction is known in detail [2], [16].

In agreement with literature data indicating that the constitutive expression of CYP1A1 is negligible in liver, while CYP1A2 is predominantly a hepatic enzyme [26]–[28], the qPCR determinations of this study show that CYP1A1 mRNA is virtually absent in the liver of uninduced rats, whereas CYP1A2 mRNA is present at a significant level in control healthy rats. Consistent with the results of qPCR determinations, the Western blot analysis shows that no CYP1A1 protein is detectable in uninduced rats, whereas CYP1A2 protein is constitutively expressed. The results of both Western blot and qPCR analyses also show that basal mRNA and protein expressions of CYP1A2 decrease significantly in cirrhotic rats in proportion to the severity of liver cirrhosis (by about 45 and 80% in non-ascitic and ascitic rats, respectively). This finding is in good agreement with human data indicating that CYP1A2 protein expression decreases on average by 71% in patients with severe hepatocellular cirrhosis [29].

Following BP treatment, mRNA and protein expressions of both CYP1A enzymes are dramatically increased to very similar levels in healthy and non-ascitic rats, confirming previous indications that inducibility is essentially preserved in the compensated state of cirrhosis [11]–[13]. On the contrary, the results of qPCR and Western blot analyses show a markedly reduced increase in the mRNA level and protein expression of CYP1A1 and CYP1A2 in ascitic rats, indicating that induction is greatly curtailed in decompensated cirrhosis.

There is a general consensus that the transcription of CYP1A enzymes is increased by BP *via* AhR activation [2], [16], and it has been shown that AhR expression is up-regulated following the administration of various AhR ligands [21], [30], [31]. Therefore, in an attempt to explain the much lower increase in mRNA levels of CYP1A enzymes in induced ascitic rats, both basal and induced mRNA and protein expressions of AhR were determined. The results show an apparent dissociation between the basal levels of AhR mRNA, which are similar in the three groups of rats, and the corresponding protein expression, which is significantly lower in ascitic rats. Such a dissociation between mRNA and protein expression has been observed with various proteins including CYP enzymes, and has been ascribed to translational repression by microRNAs [32], some of which are overexpressed in proportion to the degree of liver fibrosis [33]. However, following BP induction, both the mRNA level and the protein expression of AhR in ascitic rats are significantly lower compared with healthy and non-ascitic animals. Thus, it cannot be excluded that differences in basal mRNA levels of AhR could not be detected due to their low constitutive expression. Whatever the explanation may be, our results show that BP treatment increases both the mRNA level and protein expression of AhR to a significantly lower level in ascitic rats, thereby providing a plausible explanation for the much lower inducing effect of BP on mRNA and protein expressions of CYP1A enzymes in this group of animals. However, the possibility cannot be ruled out that a cirrhosis-associated decrease in the expression of Arnt, with which AhR dimerizes [2], [16], or other AhR cofactors may concur in decreasing the inducibility of CYP1A enzymes in severe liver dysfunction.

Previous studies of CYP1A-catalyzed enzymatic activities [21], [25] have shown that EROD activity, although currently used as a marker reaction for CYP1A1, is only specific in rats induced by means of AhR ligands, whereas it is catalysed by other CYPs (mainly CYP2C6) in uninduced rats, in which (as this study also shows; Fig. 4), CYP1A1 is virtually absent. Consistent with the observations of these previous studies [21], [25], our kinetic analysis shows that the K_m_ value of EROD activity in uninduced animals is greater than 1 µM (results not shown), i.e. more than 5 times higher than that of BP-induced EROD activity (0.20 µM, Table 2), thereby confirming that different CYPs catalyze EROD activity in control and BP-treated animals. For this reason, we did not report EROD activity of uninduced animals. At variance with EROD, MROD activity has been shown to be a specific marker reaction for CYP1A2 in both uninduced and BP-treated rats [21], [25]. The results of our kinetic analysis are in keeping with those of qPCR and Western blot experiments, since they show that induced EROD (CYP1A1) activity is totally preserved in rats with non-ascitic cirrhosis, whereas it is markedly reduced (by about 60%) in ascitic rats. The effect of cirrhosis on basal and induced MROD (CYP1A2) activities is also similar to that observed on mRNA and protein expression of CYP1A2 since, a) as the severity of liver dysfunction increases, basal MROD activity decreases to a level as low as 30% of that in healthy animals; b) the induction of MROD activity is preserved in rats with non-ascitic cirrhosis, whereas it is drastically reduced (by more than 70%) in ascitic rats.

In conclusion, our methodological approach, involving the evaluation of rats rigorously stratified according to the degree of liver dysfunction, has enabled us to provide conclusive evidence that enzyme inducibility is still preserved in the compensated state of cirrhosis, whereas it is drastically reduced when liver dysfunction becomes severe. This finding may at least in part explain why conflicting results were obtained by human studies, which examined patient populations with different degrees of liver insufficiency [10]. Moreover, the simultaneous assessment of mRNA and protein expressions of CYP1A enzymes and AhR has, at least in part, clarified the mechanism responsible for the cirrhosis-associated decrease of inducibility. In particular, the observation that induced mRNA level, protein expression and enzymatic activity are proportionally decreased in ascitic rats indicates that the process of enzyme induction is compromised at the pretranslational level. Although, in principle, decreased mRNA levels can be the result of either decreased mRNA transcription or increased mRNA turnover, the finding that both constitutive and induced protein expressions of AhR are decreased in rats with ascitic cirrhosis appears to be consistent with reduced transcription of CYP1A genes. This finding may also explain the cirrhosis-associated decrease in the constitutive level of CYP1A2 mRNA (Fig. 3B), as previously observed also in human beings [34].

Since various different mechanisms mediate CYP induction, the above conclusions regarding CYP1A enzymes cannot *a priori* be extended to the induction of all CYP forms. Therefore, there may be a possibility that, like the constitutive expression of CYP enzymes [28], [35], [36], their inducibility is differentially affected by liver dysfunction. In order to gain further insight into this question, studies are in progress in our laboratory that aim to assess the effect of liver cirrhosis on CYP induction mediated by other nuclear receptors.

## References

[1] Tucker GT, Houston JB, Huang SM (2001) Optimizing drug development: strategies to assess drug metabolism/transporter interaction potential-toward a consensus. Clin Pharmacol Ther 70: 103–114. 11503003 10.1067/mcp.2001.116891

[2] Park BK, Kitteringham NR, Pirmohamed M, Tucker GT (1996) Relevance of induction of human drug-metabolizing enzymes: pharmacological and toxicological implications. Br J Clin Pharmacol 41: 477–491. 8799511 10.1046/j.1365-2125.1996.03482.x PMC2042620

[3] Fuhr U (2000) Induction of drug metabolising enzymes: pharmacokinetic and toxicological consequences in humans. Clin Pharmacokinet 38: 493–504. 10885586 10.2165/00003088-200038060-00003

[4] Orlando R, Piccoli P, De Martin S, Padrini R, Floreani M, et al (2004) Cytochrome P450 1A2 is a major determinant of lidocaine metabolism in vivo: effects of liver function. Clin Pharmacol Ther 75: 80–88. 14749694 10.1016/j.clpt.2003.09.007

[5] Orlando R, Padrini R, Perazzi M, De Martin S, Piccoli P, et al (2006) Liver dysfunction markedly decreases the inhibition of cytochrome P450 1A2-mediated theophylline metabolism by fluvoxamine. Clin Pharmacol Ther 79: 489–499. 16678550 10.1016/j.clpt.2006.01.012

[6] Orlando R, De Martin S, Pegoraro P, Quintieri L, Palatini P (2009) Irreversible CYP3A inhibition accompanied by plasma protein-binding displacement: a comparative analysis in subjects with normal and impaired liver function. Clin Pharmacol Ther 85: 319–326. 19020496 10.1038/clpt.2008.216

[7] Palatini P, Orlando R, De Martin S (2010) The effect of liver disease on inhibitory and plasma protein-binding displacement interactions: an update. Expert Opin Drug Metab Toxicol 6: 1215–1230. 20604736 10.1517/17425255.2010.503704

[8] Hoyumpa AM Jr, Schenker S (1982) Major drug interactions: effect of liver disease, alcohol, and malnutrition. Annu Rev Med 33: 113–149. 7044269 10.1146/annurev.me.33.020182.000553

[9] Elbekai RH, Korashy HM, El-Kadi AO (2004) The effect of liver cirrhosis on the regulation and expression of drug metabolizing enzymes. Curr Drug Metab 5: 157–167. 15078193 10.2174/1389200043489054

[10] Palatini P, De Martin S, Pegoraro P, Orlando R (2008) Enzyme inhibition and induction in liver disease. Curr Clin Pharmacol 3: 56–69. 18690879 10.2174/157488408783329896

[11] Farrell GC, Zaluzny L (1984) Microsomal protein synthesis and induction of cytochrome P-450 in cirrhotic rat liver. Aust J Exp Biol Med Sci 62: 291–301. 6497779 10.1038/icb.1984.29

[12] Wu ZQ, Piché D, Vallières S, Huet PM, Gascon-Barré M (1991) Unimpaired induction of drug-metabolizing enzymes in hepatocytes isolated from rats with micronodular cirrhosis. Can J Physiol Pharmacol 69: 426–436. 2059906 10.1139/y91-065

[13] Marshall WJ, McLean AE (1969) The effect of cirrhosis of the liver on microsomal detoxications and cytochrome P-450. Br J Exp Pathol 50: 578–583. 5364388 PMC2072168

[14] Jiménez W, Martinez-Pardo A, Arroyo V, Bruix J, Rimola A, et al (1985) Temporal relationship between hyperaldosteronism, sodium retention and ascites formation in rats with experimental cirrhosis. Hepatology 5: 245–250. 3979957 10.1002/hep.1840050215

[15] Jiménez W, Clária J, Arroyo V, Rodés J (1992) Carbon tetrachloride induced cirrhosis in rats: a useful tool for investigating the pathogenesis of ascites in chronic liver disease. J Gastroenterol Hepatol 7: 90–97. 1543874 10.1111/j.1440-1746.1992.tb00940.x

[16] Nebert DW, Dalton TP, Okey AB, Gonzales FJ (2004) Role of Aryl Hydrocarbon Receptor-mediated induction of CYP1 enzymes in environmental toxicity and cancer. J Biol Chem 279: 23847–23850. 15028720 10.1074/jbc.R400004200

[17] Da Rosa DP, Bona S, Simonetto D, Zettler C, Marroni CA, et al (2010) Melatonin protects the liver and erythrocytes against oxidative stress in cirrhotic rats. Arq Gastroenterol 47: 72–78. 20520979 10.1590/s0004-28032010000100013

[18] Ishak K, Baptista A, Bianchi L, Callea F, De Groote J, et al (1995) Histological grading and staging of chronic hepatitis. J Hepatol 22: 696–699. 7560864 10.1016/0168-8278(95)80226-6

[19] Pearce RE, McIntyre CJ, Madan A, Sanzgiri U, Draper AJ, et al (1996) Effects of freezing, thawing, and storing human liver microsomes on cytochrome P450 activity. Arch Biochem Biophys 331: 145–169. 8660694 10.1006/abbi.1996.0294

[20] Omura T, Sato R (1964) The carbon monoxide-binding pigment of liver microsomes. I. Evidence for its hemoprotein nature. J Biol Chem 239: 2370–2378. 14209971

[21] Floreani M, Gabbia D, Barbierato M, De Martin S, Palatini P (2012) Differential inducing effect of benzo[a]pyrene on gene expression and enzyme activity of cytochromes P450 1A1 and 1A2 in Sprague-Dawley and Wistar rats. Drug Metab Pharmacokinet 27: 640–652. 22785257 10.2133/dmpk.dmpk-12-rg-035

[22] Laemmli UK (1970) Cleavage of structural proteins during the assembly of the head of bacteriophage T4. Nature 227: 680–685. 5432063 10.1038/227680a0

[23] \23. Hanioka N, Tatarazako N, Jinno H, Arizono K, Ando M (2000) Determination of cytochrome P450 1A activities in mammalian liver microsomes by high-performance liquid chromatography with fluorescence detection. J Chromatogr B Biomed Sci Appl 744: 399–406. 10993529 10.1016/s0378-4347(00)00278-4

[24] Copeland RA (2000) Enzymes. A Practical Introduction to Structure, Mechanisms and Data Analysis. New York: John Wiley and Sons, Inc., 397 p.

[25] Burke MD, Thompson S, Weaver RJ, Wolf CR, Mayer RT (1994) Cytochrome P450 specificities of alkoxyresorufin O-dealkylation in human and rat liver. Biochem Pharmacol 48: 923–936. 8093105 10.1016/0006-2952(94)90363-8

[26] Rendic S, Di Carlo FJ (1997) Human cytochrome P450 enzymes: a status report summarizing their reactions, substrates, inducers, and inhibitors. Drug Metab Rev 29: 413–580. 9187528 10.3109/03602539709037591

[27] Shimada T, Yamazaki H, Mimura M, Wakamiya N, Ueng WF, et al (1996) Characterization of microsomal cytochrome P450 enzymes involved in the oxidation of xenobiotic chemicals in human and fetal livers and adult lungs. Drug Metab Dispos 24: 515–522. 8723730

[28] Murray GI, Melvin WT, Greenlee WF, Burke MD (2001) Regulation, function, and tissue-specific expression of cytochrome P450 CYP1B1. Annu Rev Pharmacol Toxicol 41: 297–316. 11264459 10.1146/annurev.pharmtox.41.1.297

[29] George J, Murray M, Byth K, Farrell GC (1995) Differential alterations of cytochrome P450 proteins in livers from patients with severe chronic liver disease. Hepatology 21: 120–128. 7806144

[30] Lindros KO, Oinonen T, Johansson I, Ingelman-Sundberg M (1997) Selective centrilobular expression of the aryl hydrocarbon receptor in rat liver. J Pharmacol Exp Ther 280: 506–511. 8996235

[31] Franc MA, Pohjanvirta R, Tuomisto J, Okey AB (2001) In vivo up-regulation of aryl hydrocarbon receptor expression by 2,3,7,8-tetrachlorodibenzo-p-dioxin (TCDD) in a dioxin-resistant rat model. Biochem Pharmacol 62: 1565–1578. 11755109 10.1016/s0006-2952(01)00820-6

[32] Singh D, Kashyap A, Pandey RV, Saini KS (2011) Novel advances in cytochrome P450 research. Drug Discov Today 16: 793–799. 21864709 10.1016/j.drudis.2011.08.003

[33] Wang XW, Heegaard NH, Orum H (2012) MicroRNAs in liver disease. Gastroenterology 142: 1431–1443. 22504185 10.1053/j.gastro.2012.04.007 PMC6311104

[34] George J, Liddle C, Murray M, Byth K, Farrell GC (1995) Pre-translational regulation of cytochrome P450 genes is responsible for disease-specific changes of individual P450 enzymes among patients with cirrhosis. Biochem Pharmacol 49: 873–881. 7741759 10.1016/0006-2952(94)00515-n

[35] Farrell G (1999) Effects of disease on expression and regulation of CYPs. Mol Aspects Med 20: 55–70. 10575652

[36] Verbeeck RK (2008) Pharmacokinetics and dosage adjustment in patients with hepatic dysfunction. Eur J Clin Pharmacol 64: 1147–1161. 18762933 10.1007/s00228-008-0553-z

